# Impacts of neonicotinoid use on long-term population changes in wild bees in England

**DOI:** 10.1038/ncomms12459

**Published:** 2016-08-16

**Authors:** Ben A. Woodcock, Nicholas J. B. Isaac, James M. Bullock, David B. Roy, David G. Garthwaite, Andrew Crowe, Richard F. Pywell

**Affiliations:** 1 NERC Centre for Ecology and Hydrology, Wallingford, Oxfordshire OX10 8BB, UK; 2 FERA Science Ltd., Sand Hutton, York YO41 1LZ, UK

## Abstract

Wild bee declines have been ascribed in part to neonicotinoid insecticides. While short-term laboratory studies on commercially bred species (principally honeybees and bumblebees) have identified sub-lethal effects, there is no strong evidence linking these insecticides to losses of the majority of wild bee species. We relate 18 years of UK national wild bee distribution data for 62 species to amounts of neonicotinoid use in oilseed rape. Using a multi-species dynamic Bayesian occupancy analysis, we find evidence of increased population extinction rates in response to neonicotinoid seed treatment use on oilseed rape. Species foraging on oilseed rape benefit from the cover of this crop, but were on average three times more negatively affected by exposure to neonicotinoids than non-crop foragers. Our results suggest that sub-lethal effects of neonicotinoids could scale up to cause losses of bee biodiversity. Restrictions on neonicotinoid use may reduce population declines.

## Abstract (web summary)

Neonicotinoid as insecticide on oilseed rape can reduce bee colony density, but its effect at a large geographical scale is unclear. This study describes 18-year long wild bee tracking data in England and show neonicotinoid use is correlated with wild bee population declines at real landscape scales.

## Graphical abstract

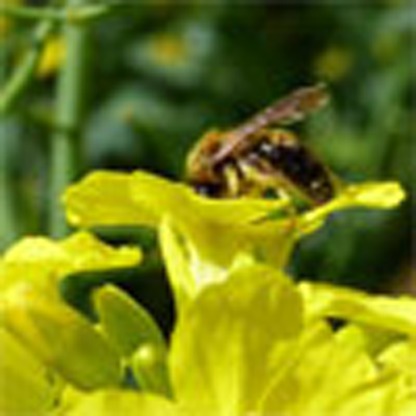

Insect pollinators are estimated to support 9.5% of world food production^1^ and wild bees have an important role in the delivery of this ecosystem service^2^. However, wild bees have undergone global declines that have been linked to habitat loss and fragmentation, pathogens, climate change and insecticides^3^,^4^,^5^,^6^,^7^. Recent debate about causal factors has focused on the role of neonicotinoid insecticides that are used worldwide as seed dressings to control pests of economically important crops^8^,^9^,^10^. The active compound of these insecticides is expressed systemically throughout plant tissues, leading to potential ingestion where honeybees^8^ and wild bees^9^,^10^,^11^,^12^ feed on the pollen and nectar of treated crops. The exposure risk to pollinators is large; in 2008 neonicotinoids comprised 80% of the worldwide insecticide seed treatment market (24% of the total insecticide market)^13^.

The primary evidence for detrimental impacts of neonicotinoids is from small-scale or short-term exposure studies on bees that are commercially bred and thus suitable as model systems, principally honeybees (*Apis mellifera* L.), some bumblebees (notably *Bombus terrestris* L.) and solitary bees of the genus *Osmia* (for example, *O. bicornis* L.)^8^,^9^,^10^,^12^. Such studies have identified an 85% drop in queen production in *B. terrestris*^9^ and a 50% reduction in the reproductive output of *O. bicornis*^14^ following exposure to neonicotinoids. A recent large-scale field study conducted over a single year also identified negative impacts on the colony growth rate of *B. terrestris* and reductions in the densities of breeding *O. bicornis*^12^. In 2013, the European Union imposed a 2-year moratorium on the use of neonicotinoids to protect both domesticated and wild bees. This moratorium is scheduled to be formally reviewed in 2016, although exemptions to this ban have already been implemented in the UK.

While there is considerable experimental evidence for short-term sub-lethal effects of exposure to neonicotinoids for a few bee species, it remains unknown whether these findings can explain large-scale and long-term changes in wild bee distributions and community structure. The short-term nature of these experiments means that while they are appropriate for determining potential drivers of change and exploring underlying causal mechanisms, they cannot determine whether a particular driver is linked to bee declines over time scales relevant to population level processes^15^. However, long-term and spatially explicit distributional data exist in the UK and are suitable for addressing this question. These data have been collected mostly through volunteer surveys by skilled naturalists and collated by the Bees, Ants and Wasps Recording Society (http://www.bwars.com/). The data cover time scales relevant to population-level processes and are suited to understanding the impacts of historic changes in agricultural management.

This study tests whether commercial use of neonicotinoids on oilseed rape crops in England can be linked to bee declines in the wild at a national scale. Oilseed rape (*Brassica napus* L.) is one of the principal crops treated with neonicotinoids worldwide and is the main arable crop on which bees actively forage in the UK: the crop covers 8.2 million ha in Europe (34.1 million ha worldwide). We test the hypothesis that spatial and temporal variation in exposure to neonicotinoids applied to commercial oilseed rape crops was correlated with population extinctions of wild bees foraging on this crop. Our results provide the first evidence that sub-lethal impacts of neonicotinoid exposure can be linked to large-scale population extinctions of wild bee species, with these effects being strongest for species that are known to forage on oilseed rape crops. These results support the findings of previous studies on commercially bred pollinators that have identified the underlying mechanisms affecting mortality. This study extends existing evidence from a limited number of model species to the wider community of bees found in agricultural landscapes. These findings provide an important contribution to the evidence base underpinning the current moratorium on the use of this insecticide in the European Union.

## Results

### Multi-species dynamic Bayesian occupancy models

**Figure 1:**
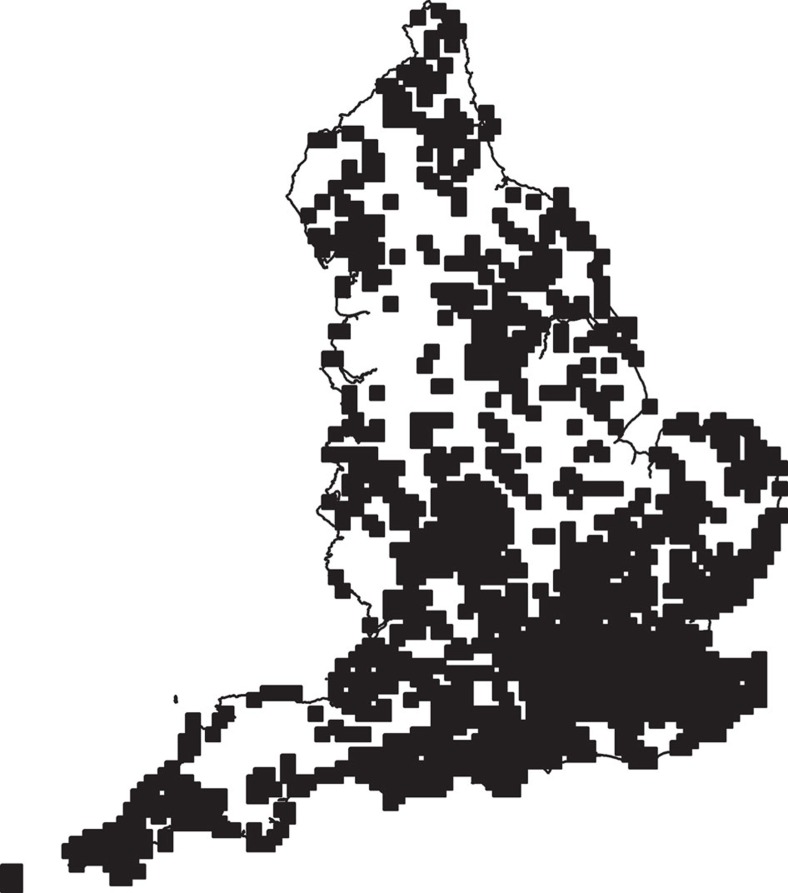
The grid cells from which bee species distributional data were derived. These were used to assess the response of individual species to oilseed rape cover, neonicotinoid exposure and the FII index. All data were derived from the Bees, Ants and Wasps Recording Scheme. Scotland and Ireland were not included in the analysis.

We constructed a multi-species dynamic Bayesian occupancy model^16^,^17^,^18^ to assess change in the occurrence of 62 wild bee species in England over a 18 year period (1994–2011). We use this model to explore the relationship between population persistence and exposure to neonicotinoid-treated oilseed rape over this period. This time period was centered on the first wide-scale commercial use of neonicotinoid seed treatments on oilseed rape in 2002. This model included spatially and temporally explicit information describing the cover of oilseed rape^19^, the area of the crop treated with neonicotinoids^20^ and an index of the combined toxicity of all foliar-applied insecticides (referred to as the foliar insecticide impact (FII) index). Note that although the FII index includes a small number of neonicotinoid based foliar applied insecticides, their non-systemic mechanism of action makes their incorporation into this index appropriate. The model used in this analysis was hierarchical and incorporates an observation sub-model that accounts for bias associated with volunteer-collected data^21^,^22^. We restricted our analysis to 1 km^2^ grid cells with surveys in at least two of the 18 years to produce a final data set that contains 31,818 surveys from 4,056 km^2^, which were nested in 1,658 25 km^2^ grid cells (Fig. 1). We excluded honeybees, since these are regularly moved across landscapes by beekeepers. Our analysis included wild bee species with records on at least 500 survey visits. Finally, we tested the prediction that bees known to forage on oilseed rape would be more likely to experience population extinctions due to higher neonicotinoid exposure than species not known to forage on this crop.

### Responses to neonicotinoid seed treatments on oilseed rape

**Figure 2:**
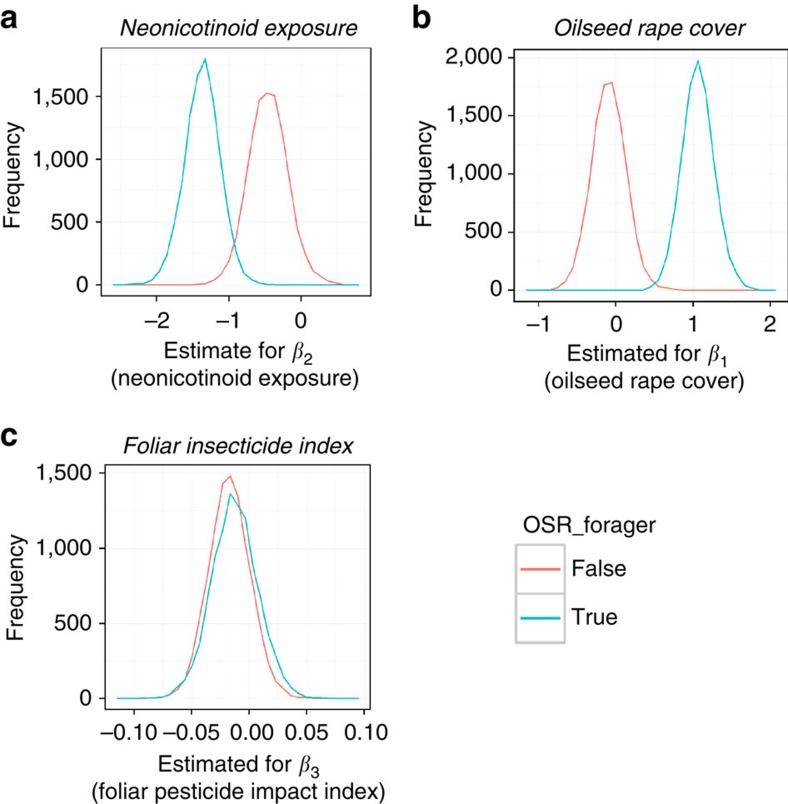
Posterior distributions for the effect sizes describing wild bee population persistence in England. The posterior distributions show the probability of parameter estimates explaining wild bee population persistence for (**a**) neonicotinoid dose rate, (**b**) oilseed rape area and (**c**) the foliar insecticide index. Posterior distributions for oilseed rape foraging and non-foraging wild bee species are shown in blue and red respectively. Mean probabilities below zero suggest negative effects of these environmental factors. Supplementary Fig. 1 provides the precision for these parameter estimates.

**Figure 3:**
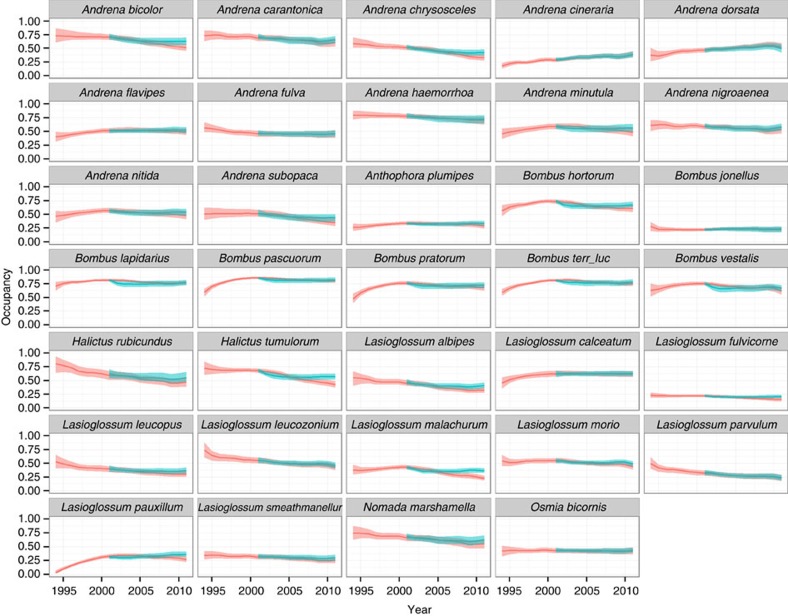
Estimates of the net effect of neonicotinoid exposure on wild bee species that forage on oilseed rape. Species population persistence trajectories are based on fitted values from individual species models (red line) and are compared with an idealized model in which no neonicotinoids were applied following their first widespread use in 2002 (blue line). Shaded areas show 95% credible intervals.

**Figure 4:**
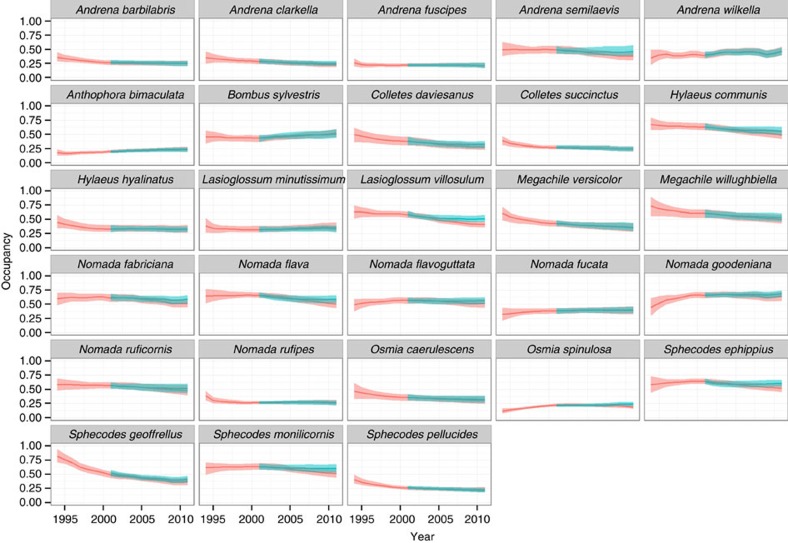
Estimates of the net effect of neonicotinoid exposure on wild bee species that do not forage on oilseed rape. Species population persistence trajectories are based on fitted values from individual species models (red line) and are compared with an idealized model in which no neonicotinoids were applied following their first widespread use in 2002 (blue line). Shaded areas show 95% credible intervals.

By grouping bees according to whether or not they forage on oilseed rape (foragers =34 species; non-foragers=28 species) we found substantial evidence for negative impacts of neonicotinoids on wild bees. Persistence was negatively correlated with neonicotinoid exposure for both oilseed rape foraging (mean=−1.37; 95% credible intervals (CI): −1.87, −0.89; >99.99% of the posterior distribution is below zero) and non-foraging species (mean=−0.46; 95% CI: −0.98, 0.09; 95.2% below zero) (Fig. 2a). The difference between the effect sizes of these two groups (0.91; 95% CI: 0.20, 1.64; 99.5% of the posterior is above zero) indicates that the negative effect of neonicotinoid exposure on persistence is three times greater for oilseed rape foragers than for non-foraging species. The difference in effect size is represented by the posterior distribution of effect sizes for oilseed rape foragers subtracted from the posterior distribution of effect sizes for non-foragers (Fig. 2a). When individual species occupancy from 1994 to 2010 was compared with occupancy predicted under the model where neonicotinoids were not used, it is clear that the detected loss of species occupancy was typically small (Figs 3 and 4). Therefore, while neonicotinoid seed treatments on oilseed rape are correlated with a reduction in population persistence for some wild bees this effect has not led to population extinction at a national scale. We estimate that neonicotinoid dose alone is responsible for a loss of greater than 20% of extant grid cells for *Halictus tumulorum*, *Lasioglossum fulvicorne*, *L. malachurum*, *L. pauxillum* and *Osmia spinulosa* since 2002 (>15% for 11 species, >10% for 24 species).

### Responses to the cover of oilseed rape

Persistence was positively correlated with oilseed rape cover (OSR) for species that forage on the crop (mean=1.06; 95% CI: 0.66, 1.49; >99.99% above zero), but not for other wild bee species (mean=−0.09; 95% CI: −0.52, 0.35; 67% below zero; Fig. 2b). This suggests that only oilseed rape foraging species benefit from the presence of oilseed rape in the landscape. Benefits of oilseed rape cover do not compensate for the negative impacts of neonicotinoid dose.

### Responses to foliar applied insecticides on oilseed rape

For groups of wild bee species we found weak negative correlations between their persistence and the application of foliar applied pesticides (foragers: mean=−0.017; 95% CI: −0.051 0.018; 83% below zero; non-foragers: mean=−0.013; 95% CI: −0.052, 0.027; 74% below zero) (Fig. 2c). The small difference in effect size between these two groups (mean=−0.004, 95% CI: −0.058, 0.049) suggests a common response to foliar applied pesticides.

### Discussion

This study provides the first evidence for community level national scale impacts on the persistence of wild bee populations resulting from exposure to neonicotinoid treated oilseed rape crops. While correlational, the identification of reduced persistence rates suggest that sublethal impacts reported by previous studies do ‘scale up' to cause population extinctions over long time scales^8^,^9^,^12^,^14^. Wild bee species that forage on oilseed rape were three times as negatively affected by exposure to neonicotinoids than non-foragers. This supports the hypothesis that the application of this pesticide to oilseed rape is a principle mechanism of exposure for wild bee communities^12^,^23^. Although not tested in the existing study this finding also suggests that other mass flowering crops (for example, sunflower) could similarly provide a route of exposure to neonicotinoids that could lead to the loss of population persistence for wild bees.

Negative correlations between population persistence and neonicotinoid exposure were also found for species not known to forage on oilseed rape. One interpretation for this is that ‘non-foraging' species have been exposed to neonicotinoids expressed in non-crop plants growing in soils contaminated with neonicotinoids. This indirect mechanisms of exposure has increasingly being identified as a potential problem in arable farming systems for wild bees^24^,^25^ and may pose a risk for species that are active outside of the flowering period of oilseed rape. An alternative, but not mutually exclusive explanation, it is some of these species may also forage on oilseed rape at a level high enough to experience reductions in population persistence, but low enough to have escaped identification as an oilseed rape forager^8^. Variation in the use of oilseed rape by different bee species is likely a common feature of wild bee communities in agricultural systems, from those that habitually forage on oilseed rape (for example, *B. terrestris*) to those that use the crop on a more opportunistic basis when other floral resources are absent^26^. Such differences in resource utilization would not only affect the risk of exposure to neonicotinoids for known foragers, but also the likelihood of identifying a species as a potential forager in the first place. However, classifying wild bees into foraging and non-foraging species based on existing observational data provided the only tractable approach for assessing exposure risk to neonicotinoids. Increased resolution in both inter-and intra-specific crop foraging preferences would improve the explanatory power of these models. It should be noted that there is also a lack of phylogenetic independence between species allocated to either oilseed rape foraging or non-foraging groups. Ultimately, the evidence from this study suggests that while there may be alternative mechanisms of exposure to neonicotinoids for wild bees (for example, soil contamination), foraging on treated oilseed rape for pollen and nectar represents the principal mechanism of exposure affecting population persistence^27^.

The short duration of flowering for oilseed rape (typically 4–6 weeks in early summer) is thought to limit the importance of this crop for many species as it is unable to provide a continuous foraging resources over the entire breeding season^28^,^29^,^30^. For example, early season foraging resources (such as those provided by oilseed rape) are important for worker production in bumblebees^23^,^29^, but current evidence suggests that queen production and subsequent population growth depends on foraging resource over the whole season^29^. However, mechanistic models demonstrate that for generalist solitary bees oilseed rape can have a positive effect on population growth^31^. Our results support these later findings with wild bee species known to forage on oilseed rape crops having increase population persistence in response to the cover of this crop. As the current analysis lacked data of sufficient resolution to assess whether the benefits of OSR were conditional on the availability of other flowering resources this finding does not dispute the importance of whole season foraging resources^28^. The spatially complex structure of English landscapes and the creation of flower rich habitats under the agri-environment schemes may mean that a sufficient continuity of foraging resources do exist that allow wild bees to benefit from the short flowering period of oilseed rape. Ultimately, the intensive nature of oilseed rape crop management, in particular its dependence on insecticides, means that its value as a foraging resource for wild bees may be outweighed by the management required to ensure crop yields^32^. However, it may be possible to cultivate oil-seed rape without extensive use of neonicotinoids: a recent UK based analysis demonstrated that on average neonicotinoid seed treatments do not boost farmer profits^32^.

Interestingly, we found that the application of foliar applied insecticides had little or no negative consequences for population persistence of wild bees. Management operations are widely implemented in English farming systems to minimize the risk of exposure for domesticated and wild bees to foliar applied insecticides. For example, codes of practice limit application times to periods of low bee activity, particularly the evening or early morning^33^. The small effect sizes for foliar applied insecticides suggest that these codes of practice may have been effective in minimizing the exposure risk for wild bees, however, it is not possible to directly test this assertion within the current analysis. Such codes of practice were developed principally to protect honeybees that forage over a well-defined daily feeding period^34^. Other wild bee species, in particular bumblebees, may forage over a larger proportion of the day and so may be more likely to suffer mortality from foliar insecticides even where codes of practice to protect them are adhered to^34^,^35^.

In conclusions, the results presented here significantly expand upon previous short-term studies by demonstrating how exposure to neonicotinoids seed treatments have impacted upon the population persistence of wild bee communities foraging on oilseed rape. Although a relatively small number of bee species typically play the dominant role in crop pollination^36^, the resilience of pollination services will often depend on the overall community^37^,^38^. Our results, therefore, have implications for the conservation of not only bee communities in intensively farmed landscapes, but the capacity of these systems to maintain stable crop pollination services in the face of changing environmental conditions^2^. These findings ultimately provide a crucial component of the evidence base needed to develop national scale management strategies that support wild bees over temporal and spatial scales that ensure population persistence over the long-term. While the evidence presented here shows that neonicotinoids were a contributing factor leading to reduced population persistence it is unlikely that its effects would act in isolation of other environmental pressures. A complex array of drivers, from land use to climatic change, may be interacting with neonicotinoid exposure in non-linear ways to affect wild bee population persistence^3^,^4^,^5^,^6^,^7^. Although not assessed in the current study, the capacity of species to recover from the impacts of neonicotinoid exposure would likely vary on an individual basis should the current moratorium on neonicotinoid use continue. However, in the absence of neonicotinoid use the benefits of oilseed rape as an early season foraging resource may mean that the recovery of at least some wild bee species may be relatively rapid.

## Methods

### Wild bee distributional data and foraging preferences

There are ∼250 species of native bees (Order Hymenoptera) known to occur in England, comprising solitary bees (for example, some Apidae, Andrenidae, Megachilidae and Halictidae), 24 species of bumblebee (Apidae: *Bombus* spp.) and the domesticated honeybee (*A. mellifera*). Approximately 20% of these species are known to act as pollinators of oilseed rape and so occur within arable farmland^37^,^39^,^40^. To assess distributional changes in these wild bee species we analysed long-term occurrence records from 1994–2011. These records were collected and verified by the Bees, Ants and Wasps Recording Society (BWARS: http://www.bwars.com/) and represent a largely volunteer-collected, national-scale distributional database that is globally unique in coverage and detail. We used only bee distributional records from England to match available data on insecticide use and oilseed rape cropping patterns. We focused on the period 1994–2011 to quantify trends both before and after the date of first use of neonicotinoids on oilseed rape in England in 2002^41^. We excluded honeybees from the current study as their hives are both artificially managed and moved around landscapes and so are not comparable with wild species^42^.

As citizen science data can be collected via wide participation projects using non-experts it has a reputation for being variable in quality. However, the UK recording schemes for invertebrates are typically more refined. Individual records are normally collected by local experts/entomologists rather than the general public (that is, who have no taxonomic experience). Under the auspices of the Bees, Wasps and Ants Recording Society, identifications are verified through photographic evidence and/or physical specimens in questionable cases. Records are also compiled centrally and subject to computer checks to identify potential outliers, such as those outside the previously known range, from atypical habitats or outside typical flight periods. In terms of the taxonomic rigour of individual records this data set is of high quality. As the data on bee distributions were collected by volunteers, not all areas are sampled with the same effort. As such while the data set is taxonomically robust there is no structured framework for how records are collected in terms of sites sampled or methods used^43^. It is for this reason that these data sets contain information only on occupancy of grid squares and not abundance. However, variation in recorder activity is a potentially significant issue in the analysis of these data sets. We use methods recommended by Isaac *et al.*^21^ to account for the effect of variation in recorder activity on trend estimation which are described in detail in the statistics section below. The data sets used here have been used as a basis for the identification of declines in pollinator species richness in the UK^4^,^44^.

### Wild bee distributional foraging preferences for oilseed rape

We classified wild bee species according to whether or not they have been observed foraging on oilseed rape in England. This was based upon published surveys from 30 English farms comprising 114 h of direct observations^7^,^8^ to produce a list of 50 bee species (including honeybees) recorded as foraging on oilseed rape (Supplementary Table 1). This list was used to classify species of bee into two categories: oilseed rape foragers and non-foragers. Due to differences in methods used to collect the data from which this list was compiled it is solely qualitative, and makes no assessment of the relative use of the crop by different species. However, the list is derived from surveys undertaken in areas of high diversity of wild bees in the UK, in particular those associated with Salisbury Plain (the largest area of pristine chalk grassland in Europe). To our knowledge this is the most comprehensive UK list of bees that forage on oilseed rape, and comprises *c.* 20% of UK bee species. All of the 20 wild bee species identified as pollinators of oilseed rape by the Kleijn *et al.*^36^ study of world crop pollinators were represented in this list, with an additional 29 wild bee species.

### Criteria for species inclusions in analysis

We converted the occurrence records into a data frame suitable for analysis by first selecting all 1 km^2^ grid cells in England with surveys in at least two-years during the period 1994–2011. We then identified all unique combinations of date and 1 km^2^ grid cell, which we henceforth refer to as a survey. Surveys with coarser spatio-temporal resolution were excluded. Our final data set contained 31,818 surveys from 4,056 1 km^2^ grid cells in England. Half the surveys were of just a single bee species, but the maximum number of species per survey was 45 species out of the total bee fauna of *c.* 250 (Fig. 1). We selected the 62 species that were recorded on at least 500 surveys from our final data set, representing 28 species of non-foraging and 34 species of oilseed rape foraging bees (Supplementary Table 2). This 500 survey threshold served two purposes. First, it excluded data poor species that could affect model convergence. Second, by including only relatively well-represented species we increased the reliability of our classification of bees as either oilseed rape foragers or non-foragers. Specifically species that may potentially have fed on oilseed rape, but due to their rarity would have been unlikely to be observed doing so, were excluded from the analysis using this threshold. In the analysis we treat *B. terrestris* and *B. lucorum* as an aggregate: workers of these species are extremely difficult to distinguishing from one another. Treating them as an aggregate avoids the possibility that our model could be biased by misidentifications, while minimizing the amount of discarded data (these are two of the commonest bees in the database).

### OSR and neonicotinoid exposure rates

Oilseed rape represents an important forage resource for many wild bees, so we hypothesized that the cover of this crop had a positive effect on population persistence^27^,^31^. This contrasted with the potentially negative impacts of exposure to neonicotinoids expressed in the pollen and nectar of pesticide-treated crop^8^,^9^,^10^,^12^. To account for this, our analysis defined two separate variables that describe both the area of oilseed rape grown and neonicotinoid exposure resulting from the treatment of that crop with neonicotinoid seed treatments. The area of sown oilseed rape was derived from the Department for Environment, Food and Rural Affairs June Survey of Agriculture and Horticulture^19^. This quantified OSR in each 5 × 5 km grid square of England from 1994 to 2010. These data were collected every two-years, so we interpolated the data values for alternate years (for example, 2005 values were set as the mean of 2004 and 2006 values).

To define the temporal and spatial changes in the exposure of wild bees to neonicotinoids we defined the extent of neonicotinoid seed treatment use as recorded by the UK Pesticide Usage Survey^45^. Neonicotinoids were widely used in oilseed rape from 2002 following the first full commercial UK licensing of this insecticide for this crop (first, Imidacloprid in 2002, followed by Clothianidin and Thiamethoxam^41^). Note, our data set includes a small number of grid cells where regulatory trials were conducted before this (1999–2001). The use of neonicotinoid seed treatments rose rapidly from 37.4% (s.e.±8.0) in 2002, to 83.0% (s.e.±5.2) of the crop treated by 2011. Data on the use of neonicotinoids was collected as part of UK commitments to the EU Statistics Regulation (1185/2009/EC) by the Food and Environment Research Agency. These data are collected every two-years (and so concurrent with the crop cover data) but are derived at a considerably coarser scale of eight Department for Environment, Food and Rural Affairs regions in England (East Midlands, Eastern, London and South East, North East, North West, South West, West Midlands and Yorkshire and the Humber). The Pesticide Usage Survey is based on information provided from 1,200 surveyed farms, stratified by region and size. Surveys incorporate inbuilt anomaly checks, including verification that application rates on individual sites lie within manufacturer recommendations. For each of the eight regions standard errors for the extrapolated rates of application are derived using non-parametric bootstrapping techniques. Following regulatory requirement these standard errors must fall below 5% (ref. 46). As such this data are considered highly reliable both within and between years. Neonicotinoid exposure in each 25 km^2^ grid square (5 × 5 km) for each year was estimated as the product of the area of oilseed rape and the proportion of that crop treated with neonicotinoids in the region within which the grid square was located. We provide a discussion of issues relating to potential collinearity problems between OSR and the proportion of the crop treated with neonicotinoids in Supplementary Note 1, and show that they do not affect the general reliability of our conclusions.

### FII index

The extent of foliar insecticide use (that is, that applied as a spray as opposed to a systemically expressed seed treatment) was defined by an aggregate index describing both the application rates of foliar insecticides on oilseed rape, as well as information on their relative toxicity for bees. This FII index produces a composite estimate of the impact of foliar sprayed non-systemic insecticides (of which the most frequently used were pyrethroids) and was based on the bee component of the Environmental Impact Quotient (EIQ)^47^. The FII was defined as:


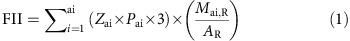


where: *Z*_ai_ is a measure of the toxicity of the active ingredient (ai) to bees. The factor 3 in equation 1 represents a weighting for comparing the relative exposure of bees to other taxa and is an integral component of the full EIQ calculation (bees and birds are given a weighting of 3, beneficial arthropods are given a weighting of 5). While redundant in the current equation it has been retained to provide consistency with the original EIQ assessment^47^. To calculate this measure of toxicity for bees, each foliar insecticide was classified by its lethal dose score (LD_50_) into high, medium or low toxicity compounds. Following established protocols^47^, high toxicity compounds (LD_50_<1 ug per bee) were given a coefficient (*Z*_ai_) of 5, medium toxicity compounds (100 ug per bee>LD_50_>1 ug per bee) a coefficient of 3 and low toxicity compounds (LD_50_>100 ug per bee) a coefficient of 1. This 5:3:1 ratio is a developed as part of the EIQ and has been widely applied in a variety of assessments of the impacts of pesticides, including studies on wild bees^48^,^49^,^50^. The variable *P*_ai_ is the plant surface half-life for active ingredient ai, which is estimated by dividing the soil deterioration half-life of the insecticide (DT_50_) by four^51^. Lethal dose toxicity (LD_50_) and soil degradation (DT_50_) data for insecticide active ingredients were derived from the Pesticide Properties Data Base^20^. *M*_ai, R_ is the mass of active ingredient applied in a region R, and *A*_R_ is the area of crop sprayed in that same region. Treating the mass and area separately was avoided to limit the potential impact of correlations between the area of oilseed rape and the pesticide pressure score. Summary data on the mass of active ingredient applied and the area treated for each region of England was derived from the Pesticide Usage Survey undertaken on alternate years^45^. Regional FII scores were calculated for each year by interpolation (as above) and assigned to each 25 km^2^ grid cell based on the region that the majority of the cell area fell within.

### Landscape structure

While landscape structure has an important role in the population persistence of many bee species^3^, its inclusion as a fixed effect in the current analysis was precluded by the absence over the 18 year period of spatially explicit data of an appropriate temporal resolution (for example, annual or biennial). However, evidence from the UK Countryside Survey undertaken in 1990, 2000 and 2007 (ref. 52) indicates there has been no significant change in the cover of Broad Habitats in England between these three time periods^52^. The main change in land use over the period has been in crop types, with the total area of cropped land and wheat cover remaining relatively constant^53^ and the cover of oilseed rape increasing largely at the cost of barley. Neither wheat nor barley are used by wild bees. It is worth noting that this survey shows that plant species richness on arable and horticulture land increased by 30% between 2000 and 2007, partly due to an increase in sown wildflower field margins which are used by wild bees^52^. Arable land therefore improved rather than declined in its quality for many wild bees over the period we study. This conclusion is supported by Carvalheiro *et al.*^44^, who show that the great loss of semi-natural habitat to agricultural intensification—which is linked to declines in wild bees—occurred before the 1990s in NW Europe.

### Statistical analysis

We created a multi-species dynamic Bayesian occupancy-detection model^18^,^54^ (BOD) to characterize distributional changes in wild bee species, implemented in the BUGS language (see Supplementary Note 2 for the BUGS code). A key feature of BOD models is that the occupancy of each grid cell (presence or absence) is separated statistically from the data collection process (detection versus non-detection): specifically, observations are conditional on the species being present. This makes BOD models well-suited to modelling change using opportunistic surveys collected by volunteers^22^, and the resulting trends are robust to multiple sources of error and bias^21^. The model we employed is ‘dynamic'^17^, in that persistence and colonization of individual grid cells is modelled explicitly (equation 2), and ‘multispecies'^16^, in that we fitted a single model to the full data set with species-specific parameter estimates. We modelled occupancy at 25 km^2^ resolution (that is, 5 × 5 km grid square) to match the spatial scale at which our covariates were calculated. In the model the expected value of z_*i,j,t*_ (occupancy of species *i* in gird square *j* in year *t*) was modelled as a function of occupancy in the previous year, z_*i,j,t-1*_. Unoccupied grid squares could be colonized with species-specific probability *γ*_*i*_, while occupied grid squares could persist from one year to the next with a probability *ϕ*_*i,j,t*._


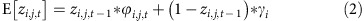


Population persistence, *ϕ*_*i,j,t*_, was modelled as a linear function of OSR, the neonicotinoid exposure and the FII in the previous year (*t−1*). Specifically:


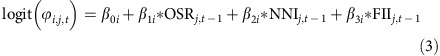


Parameter β_2*i*_ is an estimate of the annual change in the log odds ratio of the persistence from one year to the next for species *i* within the average occupied 25 km^2^ grid square. This is assessed for each unit increase in the neonicotinoid exposure; parameters *β*_1_ and *β*_3_ estimate the effect sizes of oilseed rape area and FII. Our central hypothesis is that high doses of neonicotinoids cause a reduction in population persistence (that is, *β*_2_<0).

Our detection sub-model states that the *kth* survey to a site occupied by species *i* will yield an observation with probability *p*_*i,k*_. We modelled this probability as a function of the total number of species recorded on that survey, since this provides a convenient measure of sampling effort^55^. Specifically, *p*_*i,k*_is a function of two binary variables indicating whether the survey produced a short (two or three species) or long (>3 species) list^22^:


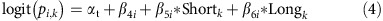


Parameter *β*_4_ is the probability that a single-species list is a survey of the focal species in the average year; parameters *β*_5_ and *β*_6_ estimate how the detection probability changes with survey effort and *α*_t_ is a random effect for year. This formulation treats short lists, long lists and single species surveys as separate data sets with different statistical properties^22^ and does not assume that all surveys record ‘complete lists' of what was present^43^. An alternative would be to use the list length as a continuous covariate on detectability^55^. However, such a monotonic function is not appropriate for bee records in the UK where a large (but unknown) proportion of records derive from casual (or ‘incidental') observations rather than formal surveys. Such incidental records disproportionately represent charismatic and easy to identify species, so that the probability of recording such a species on an incidental observation (that is, list length 1) could be higher than the probability of being recorded on a complete list derived from a short survey (list length 2–3).

Our species-specific parameter estimates treat species identity as a random effect. For parameters *γ*, *β*_0_, *β*_4_, *β*_5_, and *β*_6_ we assumed all species are drawn from a common distribution by estimating a single mean and variance for each parameter. For the covariates on population persistence (*β*_1_, *β*_2_ and *β*_3_) we assumed that species foraging on oil seed rape were drawn from a different distribution from non-foragers, following Ruiz-Gutiérrez *et al.*^16^. Comparing the posterior distributions for these groups allowed us to test the hypothesis that foragers and non-foragers respond differently, whilst fully accounting for all forms of uncertainty in the model. The covariates (OSR, neonicotinoid exposure and FII) were centered on their mean values for analysis. We ran the model described above using uninformative priors in three Monte Carlo chains of 10,000 iterations each, following a burn-in of 5,000 iterations and a thinning rate of three. We confirmed that the parameter estimates had reached convergence through a combination of quantitative (Rhat statistics^56^) and qualitative assessments (for example, visual inspection of the posterior density). We implemented the BOD model using BUGS^57^ and conducted all other analysis in the R statistical environment^58^.

There were many sites for which there were several years between surveys. In these cases, the state variable (presence-absence) was imputed following standard practice in Bayesian statistics. This imputation is likely to have smoothed our estimates of persistence (and hence occupancy) across years. Note that parameter estimates (*β*_1_, *β*_2_, and so on) were estimated over all the entire state space (that is, a large number of permutations of which sites were occupied in different years), so the posterior distributions that we derived from the model were unbiased with respect to the sparseness.

### Data availability

The wild bee distributional data that support the findings of this study are available at the BWARS data holdings accessible via the National Biodiversity Network's Gateway http://data.nbn.org.uk/ as are the Food and Environment Research Agency Pesticide Usage Survey Statistics https://secure.fera.defra.gov.uk/pusstats/myindex.cfm. The PPPB: Pesticide Properties Data Base that support the findings of this study available at http://sitem.herts.ac.uk/aeru/ppdb/en/. Finally, OSR data that support the findings of this study are available from the EDINA agcensus http://edina.ac.uk/agcensus/description.html.

## Additional information

**How to cite this article:** Woodcock, B. A. *et al.* Impacts of neonicotinoid use on long-term population changes in wild bees in England. *Nat. Commun.* 7:12459 doi: 10.1038/ncomms12459 (2016).

## Supplementary Material

Supplementary Information Supplementary Figures 1-3, Supplementary Tables 1-2, Supplementary Notes 1-2 and Supplementary References
