# Supplementary material for: Impacts of neonicotinoid use on long-term population changes in wild bees in England
Source: Nat Commun. 2016 Aug 16;7:12459. doi: 10.1038/ncomms12459 (PMC4990702; doi:10.1038/ncomms12459)
Supplement: Supplementary Information — Supplementary Figures 1-3, Supplementary Tables 1-2, Supplementary Notes 1-2 and Supplementary References [file ncomms12459-s1.pdf]

Supplementary information

Supplementary Fig 1. Precision of parameter estimates

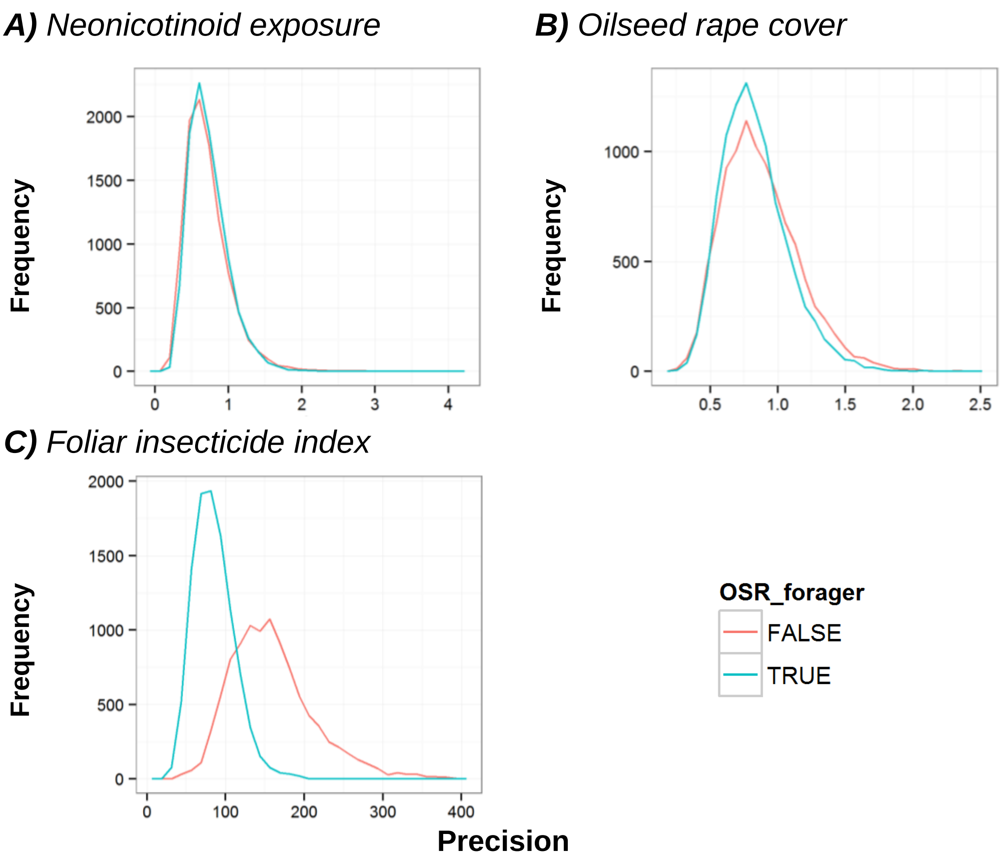

Supplementary Fig 1. The precision (1/variance across species) of the parameter estimates derived from the multi-species dynamic Bayesian occupancy-detection model. This shows the precision for oilseed rape foraging (blue) and non-foraging (red) wild bee species to the cover of neonicotinoid seed treated oilseed rape (A), the cover of oilseed rape (B), and the foliar insecticide index (C) form 1993 – 2010.

Supplementary Fig 2. Colinearity between oilseed rape cover and neonicotinoid exposure.

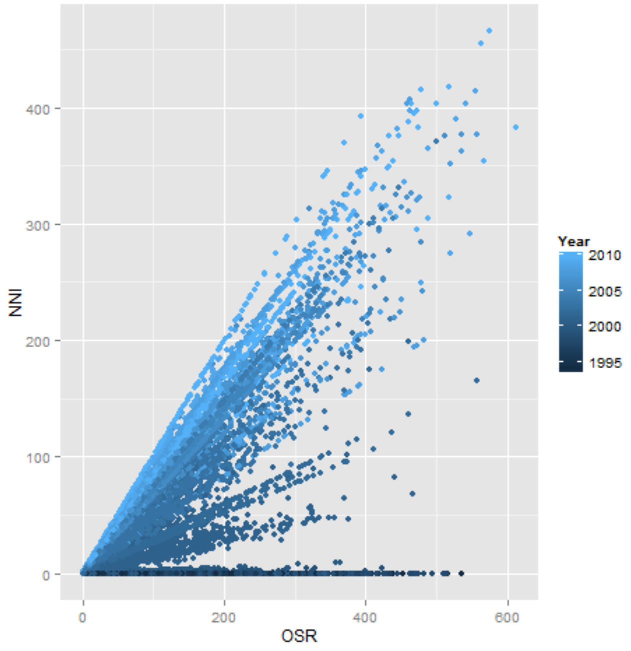

Supplementary Fig 2. Plot of raw data showing the correlations between oilseed rape cover (OSR, cover in m2 within 5 km grid squares) and neonicotinoid exposure (NNI, cover in m2 of treated crop) from 1993 to 2010.

Supplementary Fig 3. Colinearity between oilseed rape cover and neonicotinoid exposure.

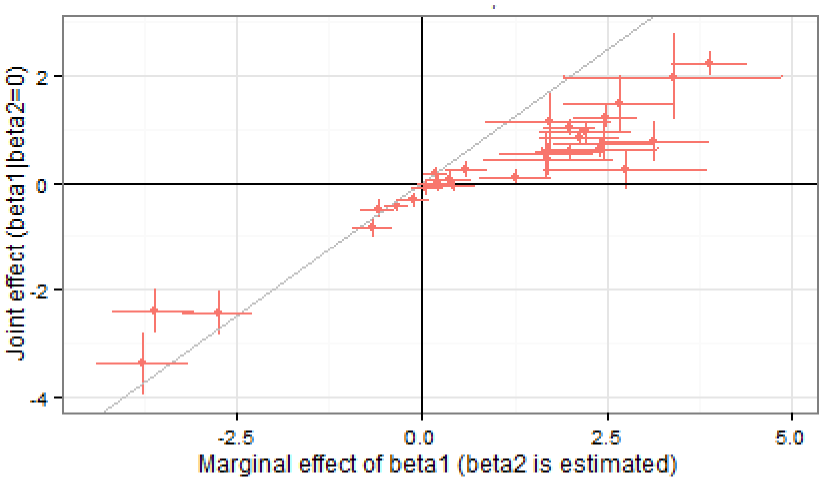

Supplementary Fig 3. Oilseed rape effect (β_1) in models with and without the neonicotinoid exposure effect (β_2). Error bars represent one standard deviation of posteriors in each direction. If collinearity was a serious problem there would be poor correlation between the estimates for β_1 (the oilseed rape effect) from the simple and complex models.

Supplementary Table 1.

Table listing species of bee (Apidae) found in association with UK oilseed rape crops. This is derived from published surveys undertaken in 2011 and 2012 [1-3] from 30 English farms and representing 114 hours of observations. It should be noted that while this is based on a robust data set it is possible that wild bee specie not recorded here may utilize the crop. With the exception of the honeybees, all species are assumed to represent un-managed wild populations. While some *Bombus* spp. (e.g. *Bombus terrestris*) may be managed as artificial colonies to promote pollination, particularly in association with glasshouse agriculture, such colonies are not typically placed into UK arable agricultural systems. Species indicated by † were identified by Kleijn et al [4] as being in the top 100 most important wild bee pollinators of world crop.

**Apidae (domesticated / managed)**
Apis mellifera Linnaeus, 1758
**Apidae (un-managed wild species)**
Anthophora plumipes (Pallas, 1772)<br>*Bombus cryptarum* (Fabricius, 1775)<br>*Bombus hortorum* (Linnaeus,1761) †<br>*Bombus hypnorum* (Linnaeus, 1758) †<br>*Bombus jonellus* (Kirby, 1802)<br>*Bombus lapidarius* (Linnaeus,1758) †<br>*Bombus lucorum* s.l. (Linnaeus, 1761) †<br>*Bombus magnus* Vogt, 1911<br>*Bombus pascuorum* (Scopoli, 1763) †<br>*Bombus pratorum* (Linnaeus, 1761) †<br>*Bombus terrestris* (Linnaeus, 1758) †<br>*Bombus vestalis* (Geoffroy,1785) *<br>*Nomada marshamella* (Kirby,1802)

* *B. vestalis* is a social parasite of *B. terrestris* and while not directly supporting its offspring by foraging on oilseed rape would though it host have larvae exposed to neonicotinoids expressed in the pollen of the crop.

Andrenidae

Andrena angustior (Kirby, 1802)
Andrena bicolor Fabricius, 1775
Andrena carantonica Pérez, 1902 †
Andrena cineraria (Linnaeus, 1758) †
Andrena chrysosceles (Kirby, 1802) †
Andrena congruens Schmiedeknecht, 1884
Andrena fucata Smith, F., 1847
Andrena dorsata (Kirby, 1802) †
Andrena fulva Müller, 1766 †
Andrena flavipes Panzer, 1799 †
Andrena haemorrhoa (Fabricius, 1781)
Andrena minutula (Kirby, 1802) †
Andrena nigrospina Thomson, 1872
Andrena nigroaenea (Kirby, 1802)
Andrena nitida (Müller, 1776) †
Andrena niveata Friese, 1887
Andrena ovatula (Kirby, 1802)
Andrena subopaca Nylander, 1848
Andrena synadelpha Perkins, 1914
Andrena helvola (Linnaeus, 1758)

Halictidae

Halictus rubicundus (Christ, 1791) †
Halictus tumulorum (Linnaeus, 1758)
Lasioglossum albipes (Fabricius, 1781)
Lasioglossum calceatum (Scopoli, 1763) †
Lasioglossum cupromicans (Pérez, 1903)
Lasioglossum fulvicorne (Kirby, 1802)
Lasioglossum leucopus (Kirby, 1802)
Lasioglossum leucozonium (Schrank, 1781)
Lasioglossum malachurum (Kirby, 1802) †
Lasioglossum morio (Fabricius, 1793) †
Lasioglossum parvulum (Schenck, 1853)
Lasioglossum pauxillum (Schenck, 1853)
Lasioglossum smeathmanellum (Kirby, 1802)
Lasioglossum zonulum (Smith, F., 1848).

Megachilidae

Osmia bicolor (Schrank, 1781)
Osmia bicornis (Linnaeus, 1758) †

Supplementary Table 2.

Table listing species of bee (Apidae) which reached the minimum threshold of data quality for inclusion in the multi-species multi-season Bayesian analysis. Species are classified as either foragers or non-foragers on oilseed rape.

**Family**	**Species**	**Oilseed rape preference**
Andrenidae	*Andrena barbilabris* (Kirby, 1802)	Non-forager
Andrenidae	*Andrena clarkella* (Kirby, 1802)	Non-forager
Andrenidae	*Andrena fuscipes* (Kirby, 1802)	Non-forager
Andrenidae	*Andrena semilaevis* Pérez,1903	Non-forager
Andrenidae	*Andrena wilkella* (Kirby,1802)	Non-forager
Andrenidae	*Andrena bicolor* Fabricius, 1775	Forager
Andrenidae	*Andrena carantonica* Pérez, 1902	Forager
Andrenidae	*Andrena chrysosceles* (Kirby, 1802)	Forager
Andrenidae	*Andrena cineraria* (Linnaeus, 1758)	Forager
Andrenidae	*Andrena dorsata* (Kirby, 1802)	Forager
Andrenidae	*Andrena flavipes* Panzer, 1799	Forager
Andrenidae	*Andrena fulva* Müller, 1766	Forager
Andrenidae	*Andrena haemorrhoa* (Fabricius, 1781)	Forager
Andrenidae	*Andrena minutula* (Kirby, 1802)	Forager
Andrenidae	*Andrena nigroaenea* (Kirby,1802)	Forager
Andrenidae	*Andrena nitida* (Müller, 1776)	Forager
Andrenidae	*Andrena subopaca* Nylander,1848	Forager
Apidae	*Anthophora bimaculata* (Panzer,1798)	Non-forager
Apidae	*Bombus sylvestris* (Lepeletier,1832)	Non-forager
Apidae	*Nomada fabriciana* (Linnaeus,1767)	Non-forager
Apidae	*Nomada flava* Panzer,1798	Non-forager
Apidae	*Nomada flavoguttata* (Kirby,1802)	Non-forager
Apidae	*Nomada fucata* Panzer,1798	Non-forager
Apidae	*Nomada goodeniana* (Kirby,1802)	Non-forager
Apidae	*Nomada ruficornis* (Linnaeus,1758)	Non-forager
Apidae	*Nomada rufipes* Fabricius, 1793	Non-forager
Apidae	*Anthophora plumipes* (Pallas, 1772)	Forager
Apidae	*Bombus hortorum* (Linnaeus,1761)	Forager
Apidae	*Bombus jonellus* (Kirby, 1802)	Forager
Apidae	*Bombus lapidarius* (Linnaeus,1758)	Forager
Apidae	*Bombus pascuorum* (Scopoli, 1763)	Forager
Apidae	*Bombus pratorum* (Linnaeus, 1761)	Forager
Apidae	*Bombus terrestris* / *luccorum* agg.	Forager
Apidae	*Bombus vestalis* (Geoffroy,1785)	Forager
Apidae	*Nomada marshamella* (Kirby,1802)	Forager

Colletidae	*Colletes daviesanus* Smith, 1846	Non-forager
Colletidae	*Colletes succinctus* (Linnaeus,1758)	Non-forager
Colletidae	*Hylaeus communis* Nylander,1852	Non-forager
Colletidae	*Hylaeus hyalinatus* Smith,1842	Non-forager
Halictidae	*Lasioglossum minutissimum* (Kirby,1802)	Non-forager
Halictidae	*Lasioglossum villosulum* (Kirby,1802)	Non-forager
Halictidae	*Sphecodes ephippius* (Linnaeus,1767)	Non-forager
Halictidae	*Sphecodes geoffrellus* (Kirby,1802)	Non-forager
Halictidae	*Sphecodes monilicornis* (Kirby,1802)	Non-forager
Halictidae	*Sphecodes pellucidus* Smith,1845	Non-forager
Halictidae	*Halictus rubicundus* (Christ,1791)	Forager
Halictidae	*Halictus tumulorum* (Linnaeus,1758)	Forager
Halictidae	*Lasioglossum albipes* (Fabricius, 1781)	Forager
Halictidae	*Lasioglossum calceatum* (Scopoli, 1763)	Forager
Halictidae	*Lasioglossum fulvicorne* (Kirby, 1802)	Forager
Halictidae	*Lasioglossum leucopus* (Kirby, 1802)	Forager
Halictidae	*Lasioglossum leucozonium* (Schrank, 1781)	Forager
Halictidae	*Lasioglossum malachurum* (Kirby, 1802)	Forager
Halictidae	*Lasioglossum morio* (Fabricius, 1793)	Forager
Halictidae	*Lasioglossum parvulum* (Schenck, 1853)	Forager
Halictidae	*Lasioglossum pauxillum* (Schenck, 1853)	Forager
Halictidae	*Lasioglossum smeathmanellum* (Kirby, 1802)	Forager
Megachilidae	*Megachile versicolor* Smith, F.,1844	Non-forager
Megachilidae	*Megachile willughbiella* (Kirby,1802)	Non-forager
Megachilidae	*Osmia caerulescens* (Linnaeus,1758)	Non-forager
Megachilidae	*Osmia spinulosa* (Kirby,1802)	Non-forager
Megachilidae	*Osmia bicornis* (Linnaeus,1758)	Forager

Supplementary Note 1. Colinearity and robustness checks

Our estimates of neonicotinoid dose incorporate information about oilseed rape cover. As such there is an inherent potential problem that collinearity may exist between two of our core response variables, i.e. oilseed rape cover and neonicotinoid dose. Oilseed rape represents the dominant mass flowering crop to which neonicotinoid seed treatments are applied within the UK (Fig. S2). As such, the correlation between neonicotinoid dose and oilseed rape is a real and unavoidable phenomenon, rather than an artefact of our treatment of the data. However, the statistical point about collinearity deserves investigation. Plotting the raw data shows that, although correlated, there is substantial variation in neonicotinoid dose that is independent of the cover of oilseed rape. In fact, the oilseed rape cover explains less than half the variation in neonicotinoid dose.

This correlation is still high enough to introduce some uncertainty into the estimates for both parameters. In technical terms, correlation in the data leads to a ridge in the likelihood surface where a range of combinations of parameter estimates are an equally good fit to the data [5]. In general, Bayesian statistics are much more robust to collinearity than Frequentist ones, because parameter estimates are expressed as credible intervals of the posterior distribution [6,7]: if serious collinearity existed then the credible intervals would be wider than in a situation where it did not exist. By contrast, frequentist statistics assume that parameter values are fixed points and standard errors on the estimates express uncertainty about the data: if collinearity exists then frequentist models are prone to false precision because the parameters cannot be separately identified [8].

One way to explore collinearity in more detail is to fit models both with and without the term for neonicotinoids (i.e. β_2 is fixed at zero). If collinearity was a serious problem, we would

find a poor correlation between the estimates for β_1 (the oilseed rape effect) from the simple and complex models. The plot (Fig. S3) shows three features. First, the correlation is tight overall: the estimate of the oilseed rape parameter (β_1) is generally similar in both sets of models. Second, the oilseed rape estimate is generally shallower when the neonicotinoid effect is absent. This is entirely expected: by omitting β_2, the estimate of β_1 is forced to explain both the positive effect of oilseed rape in addition to the negative effect of neonicotinoids. The third feature of this plot is that uncertainty (the width of the posterior) is larger in the model where β_2 is estimated than in the model where it is excluded. This additional uncertainty is due to collinearity. In other words, the simple model is able to estimate with the same precision the joint effects of oilseed rape and neonicotinoids: the complex model is able to estimate the marginal effects, but with lower precision.

In summary, we believe that potential collinearity has probably reduced the precision with which we can estimate the oilseed rape and neonicotinoid effects, but has not introduced directional bias into the parameter estimates. Therefore, the conclusions that neonicotinoid dose has a negative impact on many species, and that species foraging on oilseed rape are more negatively affected are both robust to any collinearity issue that exist.

Supplementary Note 2: Dynamic occupancy model to assess the role of neonicotinoid pesticides (and other covariates) on bees

```
#########################################################################

# Dynamic occupancy model to assess the role of neonicotinoid pesticides (and other covariates) on bees
# State model: Occupancy is a function of previous state, modified by colonization and persistence
# Persistence model: of individual grid cells, as a function of pesticide load
# Observation model: one line per visit, and DATATYPE (a measure of sampling intensity per visit)

#########################################################################

# Nick Isaac, 2015
# the state model structure comes from Royle and Dorazio [9].
# the observation part of the model is derived from van Strien et al. [10].
# the multispecies component follows Ruiz-Gutiérrez, et al. [11].
#########################################################################

model {
################################  State Priors

# for species terms where all species are drawn from a common distribution
for(i in 1:nspecies){
  # FIXED EFFECTS
  init.occ[i] ~ dunif(0, 1) # vague prior on occupancy in year 1.
```

```
  # RANDOM EFFECTS
  alpha.phi[i] ~ dnorm(mu.alpha.phi, tau.alpha.phi)
  logitgamma[i] ~ dnorm(mu.gamma, tau.gamma) # Colonisation probabilities
  logit(gamma[i]) <- logitgamma[i]

  # EFFECTS THAT ARE RANDOM BUT DEPENDENT ON TRAIT (OSR forager)
  beta1[i] ~ dnorm(mu.beta1[OSRf[i]], tau.beta1[OSRf[i]])
  beta2[i] ~ dnorm(mu.beta2[OSRf[i]], tau.beta2[OSRf[i]])
  beta3[i] ~ dnorm(mu.beta3[OSRf[i]], tau.beta3[OSRf[i]])
}

# State model hyperpriors
mu.alpha.phi ~ dnorm(0, 0.01) #persistence intercept (expected value=0, so persistence=0.5)
mu.gamma ~ dnorm(0, 0.01)

tau.alpha.phi ~ dt(0,1,1)T(0,)
tau.gamma ~ dt(0,1,1)T(0,)

for(f in 1:2){
  #Response to OSR cover differs between forarger and non-foragers
  beta1.mean[f] ~ dunif(0,1) # parameter on the measurement scale
  mu.beta1[f] <- logit(beta1.mean[f])
  tau.beta1[f] ~ dt(0,1,1)T(0,)

  #Response to NNI differs between forarger and non-foragers
  beta2.mean[f] ~ dunif(0,1) # parameter on the measurement scale
  mu.beta2[f] <- logit(beta2.mean[f])
  tau.beta2[f] ~ dt(0,1,1)T(0,)

  #Response to neonics differs between forarger and non-foragers
  beta3.mean[f] ~ dunif(0,1) # parameter on the measurement scale
  mu.beta3[f] <- logit(beta3.mean[f])
  tau.beta3[f] ~ dt(0,1,1)T(0,)
}


################################ Observation model priors
for(i in 1:nspecies){
  dtype1.p[i] ~ dnorm(mu.d1.p, tau.lp1)
  dtype2.p[i] ~ dnorm(mu.d2.p, tau.lp2)
  dtype3.p[i] ~ dnorm(mu.d3.p, tau.lp3)
}

for (t in 1:nyear) {
  alpha.t.p[t] ~ dnorm(0, tau.lp4)
}


# observation model hyperpriors
```

```
mu.d1.p ~ dnorm(-2, 0.01) # mean species effect ilt(-2) = 0.12 (probability of being recorded
on a list of length 1) #
mu.d2.p ~ dnorm(0, 0.01)
mu.d3.p ~ dnorm(0, 0.01)

tau.lp1 ~ dt(0,1,1)T(0,)
tau.lp2 ~ dt(0,1,1)T(0,)
tau.lp3 ~ dt(0,1,1)T(0,)
tau.lp4 ~ dt(0,1,1)T(0,)

################################
# State model
for (i in 1:nspecies){
 for (j in 1:nQ){ # j loop indexes the quadrant (5km grid cell), not monad
  z[i,j,1] ~ dbern(init.occ[i])
  for (t in 2:nyear){
    # Persistence at site i in year t is a function of covariates in year t-1
    logit(phi[i,j,t]) <- alpha.phi[i] + beta1[i] * OSR[j,t-1] + beta2[i] * NNI[j,t-1] + beta3[i] *
FBI[j,t-1]

    # Dynamic Occupancy = previous occupancy, modified by persistence + colonization
    muZ[i,j,t] <- z[i,j,t-1] * phi[i,j,t] + (1 - z[i,j,t-1]) * gamma[i]

    # True occupancy z at site i in year t
    z[i,j,t] ~ dbern(muZ[i,j,t])
}}}

# Observation model: go through the visits and find the matching year and site identity
for (i in 1:nspecies){
  for(k in 1:nvisit) {
  #for each visit, find the matching site, quadrant and year identities
  logit(p[i,k]) <- alpha.t.p[Year[k]] + dtype1.p[i]  + dtype2.p[i]*DATATYPE2[k] +
dtype3.p[i]*DATATYPE3[k]

  Py[i,k]<- z[i,q[Site[k]],Year[k]] * p[i,k]  # Site refers to the monad in q[Site[k]]

  y[k,i] ~ dbern(Py[i,k])
}}

######## DERIVED PARAMETERS

# difference between OSR and non foragers
beta1.diff <- mu.beta1[1] - mu.beta1[2]
beta2.diff <- mu.beta2[1] - mu.beta2[2]
beta3.diff <- mu.beta3[1] - mu.beta3[2]

# end of model formulation
}
```

Supplementary References

1. Woodcock, B. A. *et al.* National patterns of functional diversity and redundancy in predatory ground beetles and bees associated with key UK arable crops. *J. Appl. Ecol.* **51**, 142-151 (2014).
2. Woodcock, B. A. *et al.* (*Pollinator visitation data on oilseed rape varieties*. NERC-Environmental Information Data Centre DOI: 10.5285/d7b25308-3ec7-4cff-8eed-fe20b815f964, 2014).
3. Woodcock, B. A. *et al.* Crop flower visitation by honeybees, bumblebees and solitary bees: small scale behavioural differences linked to landscape scale responses. *Agric. Ecosyst. Environ.* **171**, 1-8 (2013).
4. Kleijn, D. *et al.* Delivery of crop pollination services is an insufficient argument for wild pollinator conservation. *Nat. Commun.* **6**, 7414 (2015).
5. Morgan, B. J. T. Applied Stochastic Modelling. Second Edition (CRC Press, 2008).
6. Gelman, A. & Hill, J. Data analysis using regression and multilevel/hierarchical models. (Cambridge University Press, 2007).
7. Hille Ris Lambers, J., Aukema, B. H., Diez, J., Evans, M. & Latimer, A. in Hierarchical modelling for the environmental sciences (eds J.S. Clark & A.E. Gelfand) p59 - 73 (Oxford University Press, 2006).
8. Dormann, C. F. et al. Collinearity: a review of methods to deal with it and a simulation study evaluating their performance. *Ecography* **36,** 27-46 (2013).
9. Royle, J. A. & Dorazio, R. M. *Hierarchical Modelling and Inference in Ecology*. (Academic Press, 2008).
10. van Strien, A. J., van Swaay, C. A. M. & Termaat, T. Opportunistic citizen science data of animal species produce reliable estimates of distribution trends if analysed with occupancy models. *J. Appl. Ecol.* **50**, 1450-1458 (2013).

11. Ruiz-Gutiérrez, V., Zipkin, E. F. & Dhondt, A. A. Occupancy dynamics in a tropical bird community: unexpectedly high forest use by birds classified as non-forest species. J. Appl. Ecol. 47, 621-630 (2010).
